# Involvement of 14-3-3 Proteins in Regulating Tumor Progression of Hepatocellular Carcinoma

**DOI:** 10.3390/cancers7020822

**Published:** 2015-06-15

**Authors:** Yi-Ju Wu, Yee-Jee Jan, Bor-Sheng Ko, Shu-Man Liang, Jun-Yang Liou

**Affiliations:** 1 Institute of Cellular and System Medicine, National Health Research Institutes, 35 Keyan Road, Zhunan 350, Taiwan; E-Mails: kathy614@nhri.org.tw (Y.-J.W.); shu-man@nhri.org.tw (S.-M.L.); 2 Department of Pathology and Laboratory Medicine, Taichung Veterans General Hospital, Taichung 407, Taiwan; E-Mail: yejan@vghtc.gov.tw; 3 Department of Internal Medicine, National Taiwan University Hospital, Taipei 100, Taiwan; E-Mail: kevinkomd@gmail.com

**Keywords:** 14-3-3, apoptosis, epithelial-mesenchymal transition, hepatocellular carcinoma, migration, proliferation

## Abstract

There are seven mammalian isoforms of the 14-3-3 protein, which regulate multiple cellular functions via interactions with phosphorylated partners. Increased expression of 14-3-3 proteins contributes to tumor progression of various malignancies. Several isoforms of 14-3-3 are overexpressed and associate with higher metastatic risks and poorer survival rates of hepatocellular carcinoma (HCC). 14-3-3β and 14-3-3ζ regulate HCC cell proliferation, tumor growth and chemosensitivity via modulating mitogen-activated protein kinase (MAPK), c-Jun N-terminal kinase (JNK) and p38 signal pathways. Moreover, 14-3-3ε suppresses E-cadherin and induces focal adhesion kinase (FAK) expression, thereby enhancing epithelial-mesenchymal transition (EMT) and HCC cell migration. 14-3-3ζ forms complexes with αB-crystallin, which induces EMT and is the cause of sorafenib resistance in HCC. Finally, a recent study has indicated that 14-3-3σ induces heat shock protein 70 (HSP70) expression, which increases HCC cell migration. These results suggest that selective 14-3-3 isoforms contribute to cell proliferation, EMT and cell migration of HCC by regulating distinct targets and signal pathways. Targeting 14-3-3 proteins together with specific downstream effectors therefore has potential to be therapeutic and prognostic factors of HCC. In this article, we will overview 14-3-3’s regulation of its downstream factors and contributions to HCC EMT, cell migration and proliferation.

## 1. Introduction

14-3-3 proteins comprise seven isoforms (β, ε, γ, η, σ, τ/θ and ζ) and share highly conserved homology among all eukaryotic cells. They exert their influence by binding with Ser/Thr phosphorylated intracellular proteins, thereby affecting conformation, activity, subcellular localization and protein complex stability [1,2,3,4,5]. 14-3-3 proteins have been shown to bind a variety of proteins, including Bad, receptors, histone deacetylases (HDACs), kinases and phosphatases. Thus, 14-3-3 proteins exert influence on diverse cell functions, including cell development, cell cycle regulation, DNA repair, proliferation, apoptosis, adhesion, motility and tissue response to injury [1,2,3,4,5]. 14-3-3 proteins exhibit anti-apoptotic properties and protect cell survival through mitochondria-dependent mechanisms [3,4,5,6,7,8]. It is therefore logical to hypothesize that 14-3-3 proteins are associated with malignancies. An increasing number of reports have indicated that some 14-3-3 isoforms are overexpressed and that 14-3-3 proteins are implicated in regulating tumor progression of various types of human malignancies [9,10,11,12,13,14]. Elevated expression of selective 14-3-3 proteins is thus considered as having the potential to be tumor progression promoters.

## 2. Expression of 14-3-3 Proteins in HCC

Several studies have indicated that 14-3-3β, 14-3-3ε, 14-3-3γ, 14-3-3σ (also known as stratifin) and 14-3-3ζ isoforms are overexpressed in hepatocellular carcinoma (HCC) [15,16,17,18,19,20,21]. It was first demonstrated that increased 14-3-3ε expression is associated with a poorer overall and progression-free survival rate in HCC [15]. It is worth noting that 14-3-3ε overexpression significantly correlates with extrahepatic metastasis [15]. These results reveal that 14-3-3ε may contribute to cell survival regulation, proliferation, epithelial-mesenchymal transition (EMT) migration, as well as HCC invasion, which eventually lead to tumor growth and metastasis promotion. In addition, later studies reported that the expression of other 14-3-3 isoforms is elevated in HCC. Expression of 14-3-3γ has been implicated in promoting cell proliferation [22,23], and results from a proteomics study have revealed that 14-3-3γ is detected in HCC [24]. Increased 14-3-3β stimulates cell proliferation and tumor formation [25], whereas knockdown of 14-3-3β reduces rat hepatoma cell proliferation and tumor growth [26]. Various studies have indicated that 14-3-3β and 14-3-3γ are abundantly expressed and significantly associated with poorer survival rates and higher risks of HCC metastasis [16,17].

14-3-3ζ is overexpressed in hepatoma cell lines and in tumors of HCC patients, whereas silencing of 14-3-3ζ by RNA interference suppresses tumor cell proliferation [18]. It was reported that αB-crystallin complexes with and induces 14-3-3ζ expression, thereby activating downstream signaling to promote EMT and causing certain drug resistances in HCC [19].

The reports of 14-3-3σ expression in tumors are controversial. Earlier studies have indicated that 14-3-3σ is activated by the tumor suppressor protein p53 [27], and epigenetic silencing of 14-3-3σ by promoter methylation has been reported in various cancer cells [28,29]. In contrast, an increasing number of recent studies have demonstrated that increased expression of 14-3-3σ promotes tumor progression [30,31,32,33,34,35,36,37]. In HCC, eliminating 14-3-3σ expression by hypermethylation of CpG islands was reported in an earlier study [38]. However, increased 14-3-3σ expression in HCC was shown in later reports [15,21,39]. Taken together, these results suggest that 14-3-3 proteins are potential prognostic markers of HCC.

## 3. 14-3-3 Proteins Regulate HCC Cell Proliferation

14-3-3 proteins activate Raf-1 kinase and subsequently induce the activity of mitogen-activated protein kinase (MAPK) and extracellular regulated kinase (ERK) signaling [40,41,42]. Growth factors activate the mechanistic target of rapamycin complex 1 (mTORC1) through Akt-mediated phosphorylation of tuberous sclerosis protein 1/2 (TSC1/2), resulting in the formation of the TSC1/2 and 14-3-3 complex [43]. Thus, 14-3-3 proteins may regulate multiple cellular functions, including cell proliferation, transformation and migration, through altering phosphoinositide 3-kinase (PI3K), MAPK and mTOR signaling.

14-3-3γ is abundantly expressed, and overexpression of 14-3-3γ induces polyploidization of lung cancer cells [44]. Expression of 14-3-3γ is stimulated by IL-3, and 14-3-3γ overexpression promotes cell survival and growth by activating PI3K and MAPK signaling in hematopoietic cells [22]. These results suggest that 14-3-3γ is a crucial factor to modulate cell proliferation. Result from a proteomic study by two-dimensional difference gel electrophoresis and mass spectrometry reveals that 14-3-3γ is one of the potential biomarker of HCC [24]. A further study indicated that overexpression of 14-3-3γ is associated with extrahepatic metastasis and overall survival of HCC patients [16]. 14-3-3γ is thus considered a potential factor that contributes to HCC tumor progression by modulating cell proliferation and survival.

A previous study indicated that 14-3-3ζ is significantly overexpressed in HCC cells and tissues [18]. Suppression of 14-3-3ζ by siRNA inhibits HepG2 hepatoma cell proliferation, and depletion of 14-3-3ζ expression results in the reduction of the tumorigenicity of Huh-7 cells [18]. Besides, silencing of 14-3-3ζ affects the chemosensitivity of HCC cells by regulating JNK and p38 signaling [18]. However, the molecular mechanism for how 14-3-3ζ is involved in JNK/p38-regulating chemosensitivity remains unclear.

14-3-3β is implicated in the regulation of cell proliferation and migration. It was reported that overexpression of 14-3-3β induced cell proliferation, anchorage-independent growth and tumor growth of transformed cells via altering the activation of the MAPK signal pathway [17]. Moreover, increased 14-3-3β expression is associated with promoting cell proliferation in distinct types of cancer cells [45,46], whereas knockdown of 14-3-3β suppresses *in vitro* cell proliferation and *in vivo* tumor growth of rat hepatoma K2 cells [26]. 14-3-3β binds and forms a complex with 14-3-3β interactant 1 (FBI1) and transcriptionally suppresses the expression of MAPK phosphatase-1, thereby activating MAPK signaling to promote tumorigenicity and metastasis [47]. In addition, a further study focused on investigating the role of 14-3-3β on HCC tumor progression. Elevated 14-3-3β expression was significantly associated with higher incidence of metastasis and worse overall survival of HCC [17]. In addition, overexpression of 14-3-3β promotes HCC cell migration and proliferation, whereas treatment of the MEK-1 inhibitor abolished 14-3-3β-induced cell proliferation and migration [17]. These results suggest that 14-3-3β plays role as a promoting factor for cell proliferation of HCC. 

## 4. Anti-Apoptotic Effects of 14-3-3

14-3-3 proteins protect cells from apoptosis by binding and retaining of phosphorylated Bad. Signaling from death insults leads to translocation of Bad and Bax to the mitochondrial membrane, where they form heterodimers with the anti-apoptotic Bcl-2 or Bcl-XL, thereby disrupting mitochondrial membrane potential and inducing permeability transition [48,49,50]. Through their high binding affinity for phosphorylated Bad, 14-3-3 proteins represent a major class of cytosolic proteins that play a physiological role in controlling apoptosis via the mitochondrial pathway. It has been reported that Akt phosphorylates Bad at Ser-136, ribosomal s6 kinase (RSK) at Ser-112 and protein kinase A (PKA) at Ser-155 [51,52,53,54,55]. Results from several studies suggest that phosphorylation at Ser-155 by PKA is a pre-requisite for Bad dissociation from Bcl-XL, and the dissociated Bad is subsequently phosphorylated at Ser-112 and/or Ser-136 to facilitate its binding to 14-3-3 proteins [56].

14-3-3 also has been reported to interact with apoptosis signal resulting kinase 1 (ASK1). The ASK1 is a general mediator of cell death induced by many death stimuli, including tumor necrosis factor-α, Fas and some anticancer drugs. ASK1 specifically interacts with 14-3-3ζ via the site involving Ser-967 of ASK-1. Overexpression of 14-3-3ζ blocked ASK1-induced apoptosis, whereas disruption of ASK-1 with 14-3-3ζ interaction increased ASK1-induced apoptosis. The pro-apoptotic activity of ASK1 is antagonized by its binding to 14-3-3 proteins [57,58].

Another anti-apoptotic property of 14-3-3 proteins is attributed to their binding of phosphorylated Raf-1 and protection of Raf-1 kinase activity [40,41,42]. Raf-1 has been shown to be an important regulator of endothelial cell survival induced by vascular endothelial growth factor (VEGF) [59]. RSK is phosphorylated by ERK1/2, and RSK is involved in Raf-1/MEK-mediated cell survival signaling [60]. RSK has been shown to mediate cell survival by phosphorylating Bad at Ser-112 [60,61]. RSKs also phosphorylate transcriptional activators, such as cAMP response element-binding protein (CREB) [61]. Thus, binding of Raf-1 by 14-3-3 may play an important role in activating RSK, which, in turn, promotes survival by phosphorylating Bad and other transactivators.

## 5. 14-3-3 Proteins Contribute to HCC EMT

EMT is a complicated, but essential process for tumor metastasis progression. Loss or attenuation of E-cadherin expression serves as a hallmark for EMT, and it is frequently associated with tumor malignancy, metastasis, recurrence and patient survival rates [62,63]. E-cadherin expression is downregulated by promoter methylation [64,65] or transcriptional repressors, including Snail, Slug, Twist, Zeb-1, Zeb-2 and SIP1 [66,67,68]. The activated transcription repressors bind to the E-box motif on the promoter region, thereby suppressing the transcriptional expression of E-cadherin [66,67,68].

Overexpression of 14-3-3ε correlates with the extrahepatic metastasis of HCC [15], implying that 14-3-3ε is potentially involved in regulating cancer cell EMT, migration and invasion. 14-3-3ε overexpression affects the expression of the EMT markers. It reduces E-cadherin levels and increases vimentin and *N*-cadherin levels [69], whereas knockdown of 14-3-3ε by siRNA inhibits EMT [69]. 14-3-3ε induces Snail and Zeb-1 expression, but has no significant effect on other E-cadherin repressors [69]. Although knockdown of either Snail or Zeb-1 by siRNAs suppresses HCC cell migration, 14-3-3ε-reduced E-cadherin expression was selectively abolished by the knockdown of Zeb-1, but not of Snail [69]. These results suggest that 14-3-3ε’s regulation of EMT and cell migration is complicated and that multiple regulators or pathways are involved. 14-3-3ε is reversely correlated with E-cadherin in regards to the association of 14-3-3ε expression with clinicopathological parameters and E-cadherin levels in HCC tumors [69]. Notably, a combination of 14-3-3ε-positive and E-cadherin-negative expression is significantly associated with higher incidences of metastasis and poorer overall survival, whereas E-cadherin-positive expression has an attenuated prognostic outcome in 14-3-3ε-positive HCC patients [69]. These results support the notion that E-cadherin is a potential downstream factor regulated by 14-3-3ε expression in HCC. Thus, 14-3-3ε contributes to EMT regulation in HCC.

14-3-3 proteins were reported to interact with Snail and Ajuba in breast cancer and 293 cells [70]. Snail contains two putative 14-3-3 binding motifs, selectively interacts with 14-3-3γ, 14-3-3ε, 14-3-3τ and 14-3-3η isoforms and weakly associates with the 14-3-3β isoform [70]. These 14-3-3 isoforms interact with Snail and the co-repressor Ajuba forming a transcriptional complex that facilitates the suppression of E-cadherin, consequently promoting cancer cell EMT [70]. However, whether 14-3-3 interacts with Snail or other repressors of E-cadherin in HCC remains unclear.

Partitioning defective 3 (Par-3) is one of the crucial factors involved in modulating cell polarity, consequently affecting EMT and migration. Par-3 participates in the polarity complex and regulates cell polarity through interacting with several GTP-bound regulators [71,72,73]. Par-3 expression is increased in HCC and is associated with distant metastasis and poor overall survival rates in HCC patients [74]. 14-3-3 (also known as Par-5 in nematodes and flies) interacts with Par-3 and controls cell polarity via a phosphorylation-dependent manner [75]. Furthermore, expression of Par-3 is significantly correlated with 14-3-3ε expression [74]. Thus, 14-3-3ε may synergize with Par-3 in controlling cell polarity, resulting in promoting HCC EMT and metastasis.

It has been shown that 14-3-3ζ collaborates with ErbB2 to promote tumor progression of breast cancer via EMT induction [76]. 14-3-3ζ also promotes EMT by interacting and regulating TGFβ receptor signaling and the PI-3K subunit of p85 in breast cancer [76,77]. 14-3-3ζ also plays an important role in HCC. 14-3-3ζ is overexpressed in HCC and promotes cell proliferation, whereas knockdown of 14-3-3ζ suppressed cell proliferation via the activation of c-Jun N-terminal kinase (JNK) signaling [35]. 14-3-3ζ was identified as one of the αB-crystallin-interacting proteins [36]. αB-Crystallin belongs to the mammalian small heat shock protein superfamily, and it functions as a chaperone to protect cells from stress-induced damage [78]. Expression of αB-crystallin and 14-3-3ζ are increased in HCC, and overexpression of αB-crystallin induces EMT and enhances resistance to sorafenib via ERK signal pathway activation [36]. Moreover, αB-crystallin co-localizes and directly binds with 14-3-3ζ, consequently enhancing the stability of 14-3-3ζ and inducing Slug expression to promote HCC’s EMT process [36]. In conclusion, increased expression of selective 14-3-3 isoforms, including 14-3-3ε and 14-3-3ζ, induces specific signaling and regulator factors that facilitate EMT, cell migration and HCC tumor metastasis.

## 6. 14-3-3 Proteins Promote HCC Cell Migration

Focal adhesion kinase (FAK) is a non-receptor protein tyrosine kinase associated with and activated by integrins. FAK stimulates downstream signaling to regulate cell adhesion, migration and apoptosis [79,80,81,82]. FAK is overexpressed in HCC and is a potential prognostic factor predicting worse overall survival rates and higher incidences of metastasis [83]. It was reported that expression of FAK is dually regulated by p53 and NFκB [84,85]. Activated NFκB bound to the binding site on the FAK promoter region induces FAK expression and enhances cell migration [84,85]. Expression of FAK is tightly correlated with 14-3-3ε, and the positive expression of either 14-3-3ε or FAK is associated with the tumor size and extrahepatic metastasis of HCC [86]. 14-3-3ε induces FAK expression and promoter activity. Transiently-forced expression of 14-3-3ε induces nuclear translocation of NFκB, as well as enhancing the binding capacity of NFκB to the FAK promoter [86]. FAK is a crucial factor regulating cell migration, and 14-3-3ε promotes cell migration via the activation of the NFκB/FAK pathway in HCC.

14-3-3β has been implicated in modulating cell proliferation, migration and tumor growth in HCC [34]. Expression of 14-3-3β in HCC tumors is elevated, and its expression is significantly associated with distant metastasis, whereas only rare incidences of metastasis were found in 14-3-3β-negative HCC patients [34]. These results suggest that 14-3-3β plays an important role in promoting HCC tumor migration, invasion and metastasis. Forced expression of 14-3-3β increases HCC cell migration, proliferation, anchorage-independent growth and *in vivo* tumor growth [34]. These effects are abolished by siRNA knockdown of 14-3-3β or treatment with pharmacological inhibitors of MEK-1/2 [34]. It has been shown that 14-3-3β binds and maintains the activity of Raf-1, thereby enhancing Raf/MEK/ERK signaling [87,88]. These studies imply that 14-3-3β is a potential effector promoting HCC progression via the MAPK signal pathway.

14-3-3σ plays a “bipolar” role in HCC. Although frequent hypermethylation of 14-3-3σ was found [55], studies indicate that 14-3-3σ is increased in HCC [37,38,56]. It has been reported that expression of 14-3-3σ correlates to the histology grade and micro-vascular thrombi of HCC [37]. These results suggest that 14-3-3σ contributes to facilitating HCC cancer cell migration and invasion. Moreover, 14-3-3σ overexpression increases the expression of heat shock factor-1α (HSF-1α) and its downstream factor, HSP70, and consequently enhances cell migration. Knockdown of either HSF-1α or HSP70 eliminates 14-3-3σ-induced migration [37]. In addition, 14-3-3σ-induced HSF-1α/HSP70 expression is regulated by β-catenin. 14-3-3σ enhances β-catenin levels, and knockdown of β-catenin abrogates 14-3-3σ-induced cell migration [37]. These results suggest that regulation of the 14-3-3σ/β-catenin/HSF-1α/HSP70 cascade modulates HCC cell migration.

Intriguingly, 14-3-3σ was reported to contribute to cell invasion and metastasis in selective subtypes of breast cancer. Although the expression of 14-3-3σ is silenced in most types of breast cancer, 14-3-3σ stabilizes a soluble complex of actin and intermediate filaments to enhance cell invasion of the more aggressive and malignant breast cancers [89]. 14-3-3σ expression is significantly correlated with poor clinical outcomes of basal-like subtype breast cancer. This was seen by analyzing the association of 14-3-3σ with breast cancer clinicopathological characteristics [89]. These results suggest that 14-3-3σ is involved in cancer cell aggressiveness and tumor metastasis.

## 7. Conclusions

**Figure 1:**
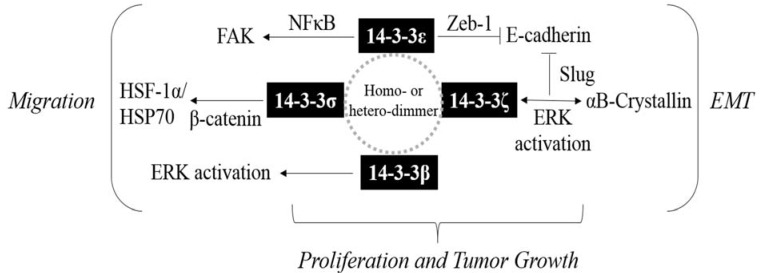
Illustrated scheme of networks for 14-3-3 and regulated downstream effectors in hepatocellular carcinoma EMT, cell migration and proliferation.

Selective 14-3-3 isoforms are overexpressed and are potential prognostic markers of HCC. 14-3-3 isoforms contribute to HCC EMT and migration/invasion and are associated with a higher risk of extrahepatic metastasis (Table 1). Overexpression of 14-3-3 proteins is thus a potential effector and can serve as a diagnostic marker for the more malignant types of HCC. 14-3-3 isoforms form homo-dimers or hetero-dimers that interact with their ligand proteins through Ser/Thr phosphorylation. As 14-3-3β, 14-3-3ε, 14-3-3σ and 14-3-3ζ isoforms are overexpressed in HCC, they may form different types of dimmers that interact with common or distinct partner proteins, thereby regulating joint or specific signaling and downstream targets. This may explain the specificity of select 14-3-3 isoform expression in different tissues or malignancies. In summary, 14-3-3ε and 14-3-3ζ contribute to HCC EMT by suppressing E-cadherin via Zeb-1 and αB-crystallin/Slug regulation, respectively. Additionally, 14-3-3β, 14-3-3γ and 14-3-3σ promote cell migration and proliferation of HCC mediated by activating Raf/MEK/ERK, PI3K/Akt signaling and increasing β-catenin/HSF-1α/HSP70 expression. The signaling pathways of JNK and p38/MAPK are involved in modulating 14-3-3ζ-associated chemosensitivity of HCC cells. Thus, 14-3-3 isoforms regulate cell proliferation, EMT and cell migration of HCC by synergistic networks to promote HCC tumor progression (Figure 1). Finally, expression of 14-3-3ζ is associated with sorafenib resistance, and 14-3-3σ is considered a potential target for drug resistance. In conclusion, expression of particular 14-3-3 isoforms is a potential prognostic marker for clinical outcomes of HCC. Targeting selective 14-3-3 isoforms and related effectors can be beneficial in developing therapeutic strategies for HCC. We therefore conclude that 14-3-3 proteins are promising prognostic markers and therapeutic targets of HCC.

**Table 1:** Studies related to 14-3-3 proteins in HCC.

Ref.	Year	Isoform	Result
Iwata *et al.* [55]	2000	14-3-3σ	Promoter of 14-3-3σ is hypermethylated, and 14-3-3σ is downregulated in HCC
Komiya *et al.* [61]	2008	14-3-3β	14-3-3β promotes tumorigenicity and metastasis in rat hepatoma
Chen *et al.* [56]	2010	14-3-3σ	14-3-3σ is detected in HCC
Ko *et al.* [32]	2011	14-3-3ε	14-3-3ε is overexpressed and associates with extrahepatic metastasis and survival of HCC
Ko *et al.* [33]	2011	14-3-3γ	14-3-3γ is overexpressed and associates with extrahepatic metastasis and survival of HCC
Liu *et al.* [34]	2011	14-3-3β	14-3-3β is overexpressed and associates with extrahepatic metastasis and survival of HCC; 14-3-3β promotes cancer cell migration, proliferation and tumor growth of HCC
Choi *et al.* [35]	2011	14-3-3ζ	14-3-3ζ is overexpressed in HCC. Silencing of 14-3-3ζ inhibits cell proliferation of HCC
Jan *et al.* [74]	2013	14-3-3ε	14-3-3ε expression is correlated with Par-3 in HCC
Liu *et al.* [69]	2013	14-3-3ε	14-3-3ε contributes to EMT in HCC cells; 14-3-3ε expression is reverse correlated with E-cadherin in HCC; a combination of 14-3-3ε/E-cadherin expression is associated with the prognosis of HCC
Huang *et al.* [36]	2013	14-3-3ζ	14-3-3ζ complexes with αB-crystallin to promote EMT and enhance resistance to sorafenib of HCC
Ko *et al.* [85]	2013	14-3-3ε	14-3-3ε induces FAK expression, and 14-3-3ε expression is correlated with FAK in HCC
Liu *et al.* [37]	2014	14-3-3σ	14-3-3σ is overexpressed and promotes cell migration of HCC
Zhang *et al.* [38]	2014	14-3-3σ	14-3-3σ is overexpressed in HCC

HCC, hepatocellular carcinoma; EMT, epithelial-mesenchymal transition.

## References

[1] Aitken A. (2011). Post-translational modification of 14-3-3 isoforms and regulation of cellular function. Semin. Cell Dev. Biol..

[2] Yang X., Lee W.H., Sobott F., Papagrigoriou E., Robinson C.V., Grossmann J.G., Sundstrom M., Doyle D.A., Elkins J.M. (2006). Structural basis for protein-protein interactions in the 14-3-3 protein family. Proc. Natl. Acad. Sci. USA.

[3] Morrison D.K. (2009). The 14-3-3 proteins: Integrators of diverse signaling cues that impact cell fate and cancer development. Trends. Cell Biol..

[4] Tzivion G., Avruch J. (2002). 14-3-3 proteins: Active cofactors in cellular regulation by serine/threonine phosphorylation. J. Biol. Chem..

[5] Tzivion G., Gupta V.S., Kaplun L., Balan V. (2006). 14-3-3 proteins as potential oncogenes. Semin. Cancer Biol..

[6] Liou J.Y., Lee S., Ghelani D., Matijevic-Aleksic N., Wu K.K. (2006). Protection of endothelial survival by peroxisome proliferator-activated receptor-δ mediated 14-3-3 upregulation. Arterioscler. Thromb. Vasc. Biol..

[7] Liou J.Y., Ghelani D., Yeh S., Wu K.K. (2007). Nonsteroidal anti-inflammatory drugs induce colorectal cancer cell apoptosis by suppressing 14-3-3epsilon. Cancer Res..

[8] Liou J.Y., Wu C.C., Chen B.R., Yen L.B., Wu K.K. (2008). Nonsteroidal anti-inflammatory drugs induced endothelial apoptosis by perturbing peroxisome proliferator-activated receptor-σ transcriptional pathway. Mol. Pharmacol..

[9] Li Z., Zhao J., Du Y., Park H.R., Sun S.Y., Bernal-Mizrachi L., Aitken A., Khuri F.R., Fu H. (2008). Down-regulation of 14-3-3ζ suppresses anchorage-independent growth of lung cancer cells through anoikis activation. Proc. Natl. Acad. Sci. USA.

[10] Neal C.L., Yao J., Yang W., Zhou X., Nguyen N.T., Lu J., Danes C.G., Guo H., Lan K.H., Ensor J. (2009). 14-3-3ζ overexpression defines high risk for breast cancer recurrence and promotes cancer cell survival. Cancer Res..

[11] Qi W., Liu X., Qiao D., Martinez J.D. (2005). Isoform-specific expression of 14-3-3 proteins in human lung cancer tissues. Int. J. Cancer.

[12] Liang S., Shen G., Liu Q., Xu Y., Zhou L., Xiao S., Xu Z., Gong F., You C., Wei Y. (2009). Isoform-specific expression and characterization of 14-3-3 proteins in human glioma tissues discovered by stable isotope labeling with amino acids in cell culture-based proteomic analysis. Proteomics Clin. Appl..

[13] Yang X., Cao W., Lin H., Zhang W., Lin W., Cao L., Zhen H., Huo J., Zhang X. (2009). Isoform-specific expression of 14-3-3 proteins in human astrocytoma. J. Neurol. Sci..

[14] Li D.Q., Wang L., Fei F., Hou Y.F., Luo J.M., Zeng R., Wu J., Lu J.S., Di G.H., Ou Z.L. (2006). Identification of breast cancer metastasis-associated proteins in an isogenic tumor metastasis model using two-dimensional gel electrophoresis and liquid chromatography-ion trap-mass spectrometry. Proteomics.

[15] Ko B.S., Chang T.C., Hsu C., Chen Y.C., Shen T.L., Chen S.C., Wang J., Wu K.K., Jan Y.J., Liou J.Y. (2011). Overexpression of 14-3-3ε predicts tumour metastasis and poor survival in hepatocellular carcinoma. Histopathology.

[16] Ko B.S., Lai I.R., Chang T.C., Liu T.A., Chen S.C., Wang J., Jan Y.J., Liou J.Y. (2011). Involvement of 14-3-3γ overexpression in extrahepatic metastasis of hepatocellular carcinoma. Hum. Pathol..

[17] Liu T.A., Jan Y.J., Ko B.S., Chen S.C., Liang S.M., Hung Y.L., Hsu C., Shen T.L., Lee Y.M., Chen P.F. (2011). Increased expression of 14-3-3β promotes tumor progression and predicts extrahepatic metastasis and worse survival in hepatocellular carcinoma. Am. J. Pathol..

[18] Choi J.E., Hur W., Jung C.K., Piao L.S., Lyoo K., Hong S.W., Kim S.W., Yoon H.Y., Yoon S.K. (2011). Silencing of 14-3-3ζ over-expression in hepatocellular carcinoma inhibits tumor growth and enhances chemosensitivity to cis-diammined dichloridoplatium. Cancer Lett..

[19] Huang X.Y., Ke A.W., Shi G.M., Zhang X., Zhang C., Shi Y.H., Wang X.Y., Ding Z.B., Xiao Y.S., Yan J. (2013). αB-crystallin complexes with 14-3-3ζ to induce epithelial-mesenchymal transition and resistance to sorafenib in hepatocellular carcinoma. Hepatology.

[20] Liu C.C., Jan Y.J., Ko B.S., Wu Y.M., Liang S.M., Chen S.C., Lee Y.M., Liu T.A., Chang T.C., Wang J. (2014). 14-3-3σ induces heat shock protein 70 expression in hepatocellular carcinoma. BMC Cancer.

[21] Zhang Y., Li Y., Lin C., Ding J., Liao G., Tang B. (2014). Aberrant upregulation of 14-3-3σ and ezh2 expression serves as an inferior prognostic biomarker for hepatocellular carcinoma. PLoS ONE.

[22] Ajjappala B.S., Kim Y.S., Kim M.S., Lee M.Y., Lee K.Y., Ki H.Y., Cha D.H., Baek K.H. (2009). 14-3-3γ is stimulated by il-3 and promotes cell proliferation. J. Immunol..

[23] Hong S.W., Qi W., Brabant M., Bosco G., Martinez J.D. (2008). Human 14-3-3γ protein results in abnormal cell proliferation in the developing eye of drosophila melanogaster. Cell Div..

[24] Lee I.N., Chen C.H., Sheu J.C., Lee H.S., Huang G.T., Yu C.Y., Lu F.J., Chow L.P. (2005). Identification of human hepatocellular carcinoma-related biomarkers by two-dimensional difference gel electrophoresis and mass spectrometry. J. Proteome Res..

[25] Takihara Y., Matsuda Y., Hara J. (2000). Role of the β isoform of 14-3-3 proteins in cellular proliferation and oncogenic transformation. Carcinogenesis.

[26] Sugiyama A., Miyagi Y., Komiya Y., Kurabe N., Kitanaka C., Kato N., Nagashima Y., Kuchino Y., Tashiro F. (2003). Forced expression of antisense 14-3-3β RNA suppresses tumor cell growth *in vitro* and *in vivo*. Carcinogenesis.

[27] Li Z., Liu J.Y., Zhang J.T. (2009). 14-3-3σ, the double-edged sword of human cancers. Am. J. Transl. Res..

[28] Lodygin D., Hermeking H. (2005). The role of epigenetic inactivation of 14-3-3σ in human cancer. Cell Res..

[29] Hermeking H. (2003). The 14-3-3 cancer connection. Nat. Rev. Cancer.

[30] Neupane D., Korc M. (2008). 14-3-3σ modulates pancreatic cancer cell survival and invasiveness. Clin. Cancer Res..

[31] Guweidhi A., Kleeff J., Giese N., El Fitori J., Ketterer K., Giese T., Buchler M.W., Korc M., Friess H. (2004). Enhanced expression of 14-3-3σ in pancreatic cancer and its role in cell cycle regulation and apoptosis. Carcinogenesis.

[32] Perathoner A., Pirkebner D., Brandacher G., Spizzo G., Stadlmann S., Obrist P., Margreiter R., Amberger A. (2005). 14-3-3σ expression is an independent prognostic parameter for poor survival in colorectal carcinoma patients. Clin. Cancer Res..

[33] Ide M., Saito K., Tsutsumi S., Tsuboi K., Yamaguchi S., Asao T., Kuwano H., Nakajima T. (2007). Over-expression of 14-3-3σ a in budding colorectal cancer cells modulates cell migration in the presence of tenascin-c. Oncol. Rep..

[34] Zhou W.H., Tang F., Xu J., Wu X., Feng Z.Y., Li H.G., Lin D.J., Shao C.K., Liu Q. (2011). Aberrant upregulation of 14-3-3o expression serves as an inferior prognostic biomarker for gastric cancer. BMC Cancer.

[35] Shiba-Ishii A., Noguchi M. (2012). Aberrant stratifin overexpression is regulated by tumor-associated cpg demethylation in lung adenocarcinoma. Am. J. Pathol..

[36] Radhakrishnan V.M., Jensen T.J., Cui H., Futscher B.W., Martinez J.D. (2011). Hypomethylation of the 14-3-3σ promoter leads to increased expression in non-small cell lung cancer. Gene. Chromosome. Canc..

[37] Shiba-Ishii A., Kano J., Morishita Y., Sato Y., Minami Y., Noguchi M. (2011). High expression of stratifin is a universal abnormality during the course of malignant progression of early-stage lung adenocarcinoma. Int. J. Cancer.

[38] Iwata N., Yamamoto H., Sasaki S., Itoh F., Suzuki H., Kikuchi T., Kaneto H., Iku S., Ozeki I., Karino Y. (2000). Frequent hypermethylation of cpg islands and loss of expression of the 14-3-3σ gene in human hepatocellular carcinoma. Oncogene.

[39] Chen X.L., Zhou L., Yang J., Shen F.K., Zhao S.P., Wang Y.L. (2010). Hepatocellular carcinoma-associated protein markers investigated by maldi-tof ms. Mol. Med. Rep..

[40] Tzivion G., Luo Z., Avruch J. (1998). A dimeric 14-3-3 protein is an essential cofactor for raf kinase activity. Nature.

[41] Roy S., McPherson R.A., Apolloni A., Yan J., Lane A., Clyde-Smith J., Hancock J.F. (1998). 14-3-3 facilitates ras-dependent raf-1 activation *in vitro* and *in vivo*. Mol. Cell Biol..

[42] Thorson J.A., Yu L.W., Hsu A.L., Shih N.Y., Graves P.R., Tanner J.W., Allen P.M., Piwnica-Worms H., Shaw A.S. (1998). 14-3-3 proteins are required for maintenance of Raf-1 phosphorylation and kinase activity. Mol. Cell Biol..

[43] DeYoung M.P., Horak P., Sofer A., Sgroi D., Ellisen L.W. (2008). Hypoxia regulates tsc1/2-mtor signaling and tumor suppression through redd1-mediated 14-3-3 shuttling. Genes Dev..

[44] Qi W., Liu X., Chen W., Li Q., Martinez J.D. (2007). Overexpression of 14-3-3γ causes polyploidization in h322 lung cancer cells. Mol. Carcinog..

[45] Musholt T.J., Brehm C., Hanack J., von Wasielewski R., Musholt P.B. (2006). Identification of differentially expressed genes in papillary thyroid carcinomas with and without rearrangements of the tyrosine kinase receptors ret and/or ntrk1. J. Surg. Res..

[46] Zeng Y., Li Y., Chen R.S., He X., Yang L., Li W. (2010). Overexpression of xct induces up-regulation of 14-3-3β in kaposi’s sarcoma. Biosci. Rep..

[47] Komiya Y., Kurabe N., Katagiri K., Ogawa M., Sugiyama A., Kawasaki Y., Tashiro F. (2008). A novel binding factor of 14-3-3β functions as a transcriptional repressor and promotes anchorage-independent growth, tumorigenicity, and metastasis. J. Biol. Chem..

[48] Adams J.M., Cory S. (1998). The Bcl-2 protein family: Arbiters of cell survival. Science.

[49] Cheng E.H., Wei M.C., Weiler S., Flavell R.A., Mak T.W., Lindsten T., Korsmeyer S.J. (2001). Bcl-2, Bcl-X_L_ sequester BH3 domain-only molecules preventing bax- and bak-mediated mitochondrial apoptosis. Mol. Cell.

[50] Zha J., Harada H., Osipov K., Jockel J., Waksman G., Korsmeyer S.J. (2000). BH3 domain of BAD is required for heterodimerization with BCL-X_L_ and pro-apoptotic activity. J. Biol. Chem..

[51] Datta S.R., Dudek H., Tao X., Masters S., Fu H., Gotoh Y., Greenberg M.E. (1997). AKT phosphorylation of BAD couples survival signals to the cell-intrinsic death machinery. Cell.

[52] Scheid M.P., Schubert K.M., Duronio V. (1999). Regulation of bad phosphorylation and association with Bcl-X_L_ by the MAPK/Erk kinase. J. Biol. Chem..

[53] Tan Y., Ruan H., Demeter M.R., Comb M.J. (1999). p90(RSK) blocks bad-mediated cell death via a protein kinase C-dependent pathway. J. Biol. Chem..

[54] Tan Y., Demeter M.R., Ruan H., Comb M.J. (2000). BAD Ser-155 phosphorylation regulates BAD/Bcl-XL interaction and cell survival. J. Biol. Chem..

[55] Lizcano J.M., Morrice N., Cohen P. (2000). Regulation of BAD by cAMP-dependent protein kinase is mediated via phosphorylation of a novel site, Ser155. Biochem. J..

[56] Zhou X.M., Liu Y., Payne G., Lutz R.J., Chittenden T. (2000). Growth factors inactivate the cell death promoter BAD by phosphorylation of its BH3 domain on Ser155. J. Biol. Chem..

[57] Subramanian R.R., Zhang H., Wang H., Ichijo H., Miyashita T., Fu H. (2004). Interaction of apoptosis signal-regulating kinase 1 with isoforms of 14-3-3 proteins. Exp. Cell Res..

[58] Zhang L., Chen J., Fu H. (1999). Suppression of apoptosis signal-regulating kinase 1-induced cell death by 14-3-3 proteins. Proc. Natl. Acad. Sci. USA.

[59] Alavi A., Hood J.D., Frausto R., Stupack D.G., Cheresh D.A. (2003). Role of Raf in vascular protection from distinct apoptotic stimuli. Science.

[60] Shimamura A., Ballif B.A., Richards S.A., Blenis J. (2000). Rsk1 mediates a MEK-MAP kinase cell survival signal. Curr. Biol..

[61] Bonni A., Brunet A., West A.E., Datta S.R., Takasu M.A., Greenberg M.E. (1999). Cell survival promoted by the Ras-MAPK signaling pathway by transcription-dependent and -independent mechanisms. Science.

[62] Jeanes A., Gottardi C.J., Yap A.S. (2008). Cadherins and cancer: How does cadherin dysfunction promote tumor progression?. Oncogene.

[63] Eger A., Stockinger A., Park J., Langkopf E., Mikula M., Gotzmann J., Mikulits W., Beug H., Foisner R. (2004). β-catenin and TGFβ signalling cooperate to maintain a mesenchymal phenotype after foser-induced epithelial to mesenchymal transition. Oncogene.

[64] Matsumura T., Makino R., Mitamura K. (2001). Frequent down-regulation of e-cadherin by genetic and epigenetic changes in the malignant progression of hepatocellular carcinomas. Clin. Cancer Res..

[65] Kanai Y., Ushijima S., Hui A.M., Ochiai A., Tsuda H., Sakamoto M., Hirohashi S. (1997). The e-cadherin gene is silenced by cpg methylation in human hepatocellular carcinomas. Int. J. Cancer.

[66] Yang B., Guo M., Herman J.G., Clark D.P. (2003). Aberrant promoter methylation profiles of tumor suppressor genes in hepatocellular carcinoma. Am. J. Pathol..

[67] Moreno-Bueno G., Portillo F., Cano A. (2008). Transcriptional regulation of cell polarity in EMT and cancer. Oncogene.

[68] Zeisberg M., Neilson E.G. (2009). Biomarkers for epithelial-mesenchymal transitions. J. Clin. Investig..

[69] Liu T.A., Jan Y.J., Ko B.S., Liang S.M., Chen S.C., Wang J., Hsu C., Wu Y.M., Liou J.Y. (2013). 14-3-3ε overexpression contributes to epithelial-mesenchymal transition of hepatocellular carcinoma. PLoS ONE.

[70] Hou Z., Peng H., White D.E., Wang P., Lieberman P.M., Halazonetis T., Rauscher F.J. (2010). 14-3-3 binding sites in the snail protein are essential for snail-mediated transcriptional repression and epithelial-mesenchymal differentiation. Cancer Res..

[71] Lin D., Edwards A.S., Fawcett J.P., Mbamalu G., Scott J.D., Pawson T. (2000). A mammalian par-3-par-6 complex implicated in cdc42/rac1 and apkc signalling and cell polarity. Nat. Cell Biol..

[72] Ooshio T., Fujita N., Yamada A., Sato T., Kitagawa Y., Okamoto R., Nakata S., Miki A., Irie K., Takai Y. (2007). Cooperative roles of PAR-3 and afadin in the formation of adherens and tight junctions. J. Cell Sci..

[73] Chen X., Macara I.G. (2005). Par-3 controls tight junction assembly through the rac exchange factor tiam1. Nat. Cell Biol..

[74] Jan Y.J., Ko B.S., Liu T.A., Wu Y.M., Liang S.M., Chen S.C., Wang J., Liou J.Y. (2013). Expression of partitioning defective 3 (PAR-3) for predicting extrahepatic metastasis and survival with hepatocellular carcinoma. Int. J. Mol. Sci..

[75] Hurd T.W., Fan S., Liu C.J., Kweon H.K., Hakansson K., Margolis B. (2003). Phosphorylation-dependent binding of 14-3-3 to the polarity protein Par3 regulates cell polarity in mammalian epithelia. Curr. Biol..

[76] Lu J., Guo H., Treekitkarnmongkol W., Li P., Zhang J., Shi B., Ling C., Zhou X., Chen T., Chiao P.J. (2009). 14-3-3ζ cooperates with erbb2 to promote ductal carcinoma *in situ* progression to invasive breast cancer by inducing epithelial-mesenchymal transition. Cancer Cell.

[77] Neal C.L., Xu J., Li P., Mori S., Yang J., Neal N.N., Zhou X., Wyszomierski S.L., Yu D. (2012). Overexpression of 14-3-3ζ in cancer cells activates pi3k via binding the p85 regulatory subunit. Oncogene.

[78] Parcellier A., Schmitt E., Brunet M., Hammann A., Solary E., Garrido C. (2005). Small heat shock proteins HSP27 and αB-crystallin: Cytoprotective and oncogenic functions. Antioxid. Redox Signal..

[79] McLean G.W., Carragher N.O., Avizienyte E., Evans J., Brunton V.G., Frame M.C. (2005). The role of focal-adhesion kinase in cancer—a new therapeutic opportunity. Nat. Rev. Cancer.

[80] Van Nimwegen M.J., van de Water B. (2007). Focal adhesion kinase: A potential target in cancer therapy. Biochem. Pharmacol..

[81] Wozniak M.A., Modzelewska K., Kwong L., Keely P.J. (2004). Focal adhesion regulation of cell behavior. Biochim. Biophys. Acta.

[82] Kornberg L., Earp H.S., Parsons J.T., Schaller M., Juliano R.L. (1992). Cell adhesion or integrin clustering increases phosphorylation of a focal adhesion-associated tyrosine kinase. J. Biol. Chem..

[83] Jan Y.J., Ko B.S., Hsu C., Chang T.C., Chen S.C., Wang J., Liou J.Y. (2009). Overexpressed focal adhesion kinase predicts a higher incidence of extrahepatic metastasis and worse survival in hepatocellular carcinoma. Hum. Pathol..

[84] Golubovskaya V., Kaur A., Cance W. (2004). Cloning and characterization of the promoter region of human focal adhesion kinase gene: Nuclear factor kappa b and p53 binding sites. Biochim. Biophys. Acta.

[85] Ko B.S., Chang T.C., Chen C.H., Liu C.C., Kuo C.C., Hsu C., Shen Y.C., Shen T.L., Golubovskaya V.M., Chang C.C. (2010). Bortezomib suppresses focal adhesion kinase expression via interrupting nuclear factor-kappa b. Life Sci..

[86] Ko B.S., Jan Y.J., Chang T.C., Liang S.M., Chen S.C., Liu T.A., Wu Y.M., Wang J., Liou J.Y. (2013). Upregulation of focal adhesion kinase by 14-3-3ε via nfkappab activation in hepatocellular carcinoma. Anticancer Agents Med. Chem..

[87] Fantl W.J., Muslin A.J., Kikuchi A., Martin J.A., MacNicol A.M., Gross R.W., Williams L.T. (1994). Activation of raf-1 by 14-3-3 proteins. Nature.

[88] Li S., Janosch P., Tanji M., Rosenfeld G.C., Waymire J.C., Mischak H., Kolch W., Sedivy J.M. (1995). Regulation of raf-1 kinase activity by the 14-3-3 family of proteins. EMBO J..

[89] Boudreau A., Tanner K., Wang D., Geyer F.C., Reis-Filho J.S., Bissell M.J. (2013). 14-3-3σ stabilizes a complex of soluble actin and intermediate filament to enable breast tumor invasion. Proc. Natl. Acad. Sci. USA.

